# Spontaneous Arteriocholedochal Fistula as a Cause of Life-Threatening Hemobilia in a Type I Giant Choledochal Cyst: A Case Report on an Unusual Situation Requiring Whipple’s Pancreatoduodenectomy

**DOI:** 10.7759/cureus.5441

**Published:** 2019-08-20

**Authors:** Humaid Ahmad, Madiha K Bajwa, Irfan Lutfi, Jahanzaib Haider, Shams Nadeem Alam

**Affiliations:** 1 Surgery, Dow University of Health Sciences, Karachi, PAK; 2 Surgery, Bahria University Medical & Dental College, Karachi, PAK; 3 Interventional Radiology, Shaheed Mohtarma Benazir Bhutto Medical College, Dow University of Health Sciences, Karachi, PAK

**Keywords:** hemobilia, life-threatening hemobilia, arteriocholedochal fistula, spontaneous arteriocholedochal fistula, giant choledochal cyst, todani type i choledochal cyst, whipple's pancreatoduodenectomy, emergency pancreatoduodenectomy, ascending cholangitis, angio-embolization

## Abstract

Life-threatening hemobilia is a rare cause of gastrointestinal hemorrhage. Giant choledochal cyst is also a rarely reported diagnosis. Similarly, arteriocholedochal fistulas are also rarely reported and usually occur after invasive procedures for diagnosis or treatment of hepatopancreatobiliary-related disorders. In this report, the authors describe a case of a spontaneous arteriocholedochal fistula that occurred in a giant choledochal cyst and led to life-threatening hemobilia. The patient ultimately required a Whipple’s pancreatoduodenectomy for treatment, which is again rarely undertaken as an emergency procedure. We describe the management this patient underwent and discuss the reasons why we resorted to undertake such a formidable procedure for the patient as his treatment option.

## Introduction

Life-threatening hemobilia is a rare cause of gastrointestinal bleeding with arteriocholedochal fistula being an unusual cause of hemobilia [1-4]. Arteriocholedochal fistulas have been reported after invasive procedures such as percutaneous transhepatic cholangiography, percutaneous biliary stent procedures, transhepatic biopsies; after trauma and surgery to the hepatobiliary tract; and even after liver transplant [1,2,4-7]. They are treated efficiently by angioembolization of the involved vessel leading to complete resolution in 80-100% cases [2,5,8].

Giant choledochal cyst in adults is also a rarely reported clinical scenario [9]. Choledochal cysts are classified according to the extent of involvement of the biliary tract with Todani type I involving the whole of the common bile duct and common hepatic duct [10]. As choledochal cysts are pre-malignant, treatment entails complete excision with Roux-en-Y biliary-enteric anastomosis. In cases of choledochal cysts where there is an intrahepatic component, simultaneous partial liver resection (type IVA) or liver transplant (type IVA and V) are also treatment options [11].

The authors report a case of a spontaneous arteriocholedochal fistula in a Todani type I giant choledochal cyst where life-threatening hemobilia occurred. Whereas arteriocholedochal fistulas and giant choledochal cysts have been reported separately, both presenting in one patient is an unreported finding [1,3-5,9,12]. The fact that this patient required a Whipple’s pancreatoduodenectomy (WPD) for treatment also makes this case unique.

## Case presentation

**Figure 1:**
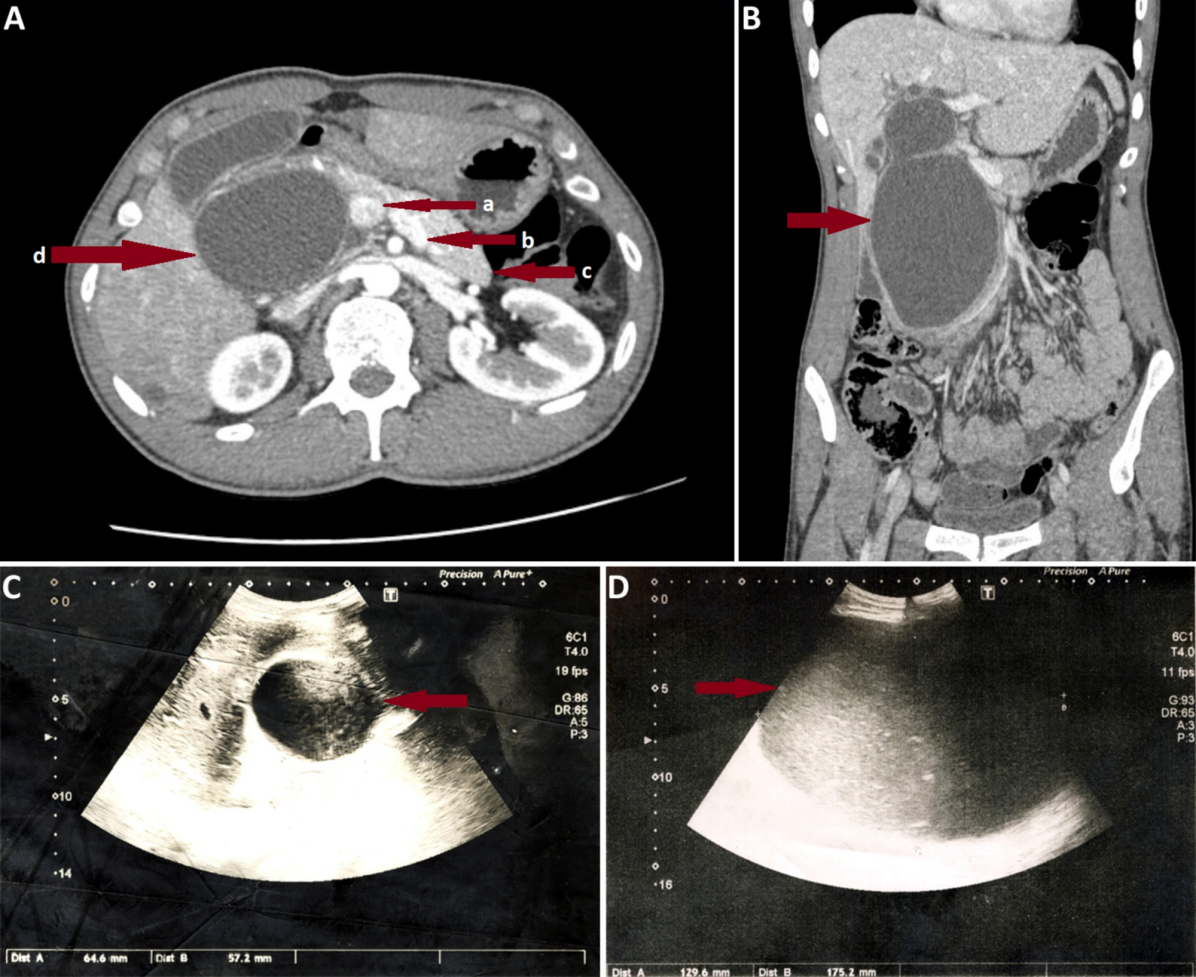
Pictures of the patient's preoperative CT scan and ultrasounds Figures 1A, 1B are axial and coronal images of preoperative CT scan of patient undertaken three weeks prior to admission, which depict the size and extent of the giant Todani type I choledochal cyst. In Figure 1A, a shows portal vein, b shows splenic vein, c shows body and tail of pancreas, and d shows the giant choledochal cyst almost completely replacing the head of the pancreas. In Figure 1B, the arrow shows the choledochal cyst in a coronal image. Figure 1C is an ultrasound image of the patient on the day of admission; the arrow shows the size of choledochal cyst. Figure 1D is an ultrasound image of the patient on the 7th-day post-admission after his condition suddenly deteriorated; the arrow shows the choledochal cyst at this point in time. In Figures 1C, 1D, the sudden increase in size on the 7th post-admission day can be appreciated.

A 25-year-old, otherwise healthy, male presented to our emergency department with severe upper abdominal pain, high-grade fever, and jaundice. He had noted a swelling in his right hypochondrium approximately two months prior to his visit. On examination, the patient was dehydrated and tachycardiac. Abdominal examination revealed a severely tender and firm mass in his right hypochondrium. A CT scan had been performed three weeks before this presentation on suspicion of an upper abdominal mass. It reported a giant Todani type I choledochal cyst (Figures 1A, 1B). On ultrasound in the emergency department, the size of the cyst was 11 cm (Figure 1C). Baseline laboratory data were significant for a total leucocyte count of 21000, total bilirubin of 6.9, and direct bilirubin of 4.6. The patient was diagnosed with ascending cholangitis due to an infected giant choledochal cyst and was placed on conservative management with broad-spectrum antibiotics. During the ensuing week, the patient’s condition improved, and the element of sepsis and jaundice settled down. The severe tenderness in his right hypochondriac mass also resolved. The patient was continued on conservative management with the plan to undertake resection of the cyst followed by Roux-en-Y biliary-enteric anastomosis on elective list.

On the 7th post-admission day, the patient developed class II hypovolemic shock with a sudden increase in the right upper abdominal pain. His also became severely anemic. Abdominal examination revealed that the mass in the right hypochondrium had become severely tender again and had suddenly grown in size and extended into the right iliac fossa. The patient was resuscitated and shifted to the High Dependency Unit. An urgent ultrasound revealed that the cyst had increased to a size of 17 cm (Figure 1D) and showed increased internal echoes that represented blood.

**Figure 2:**
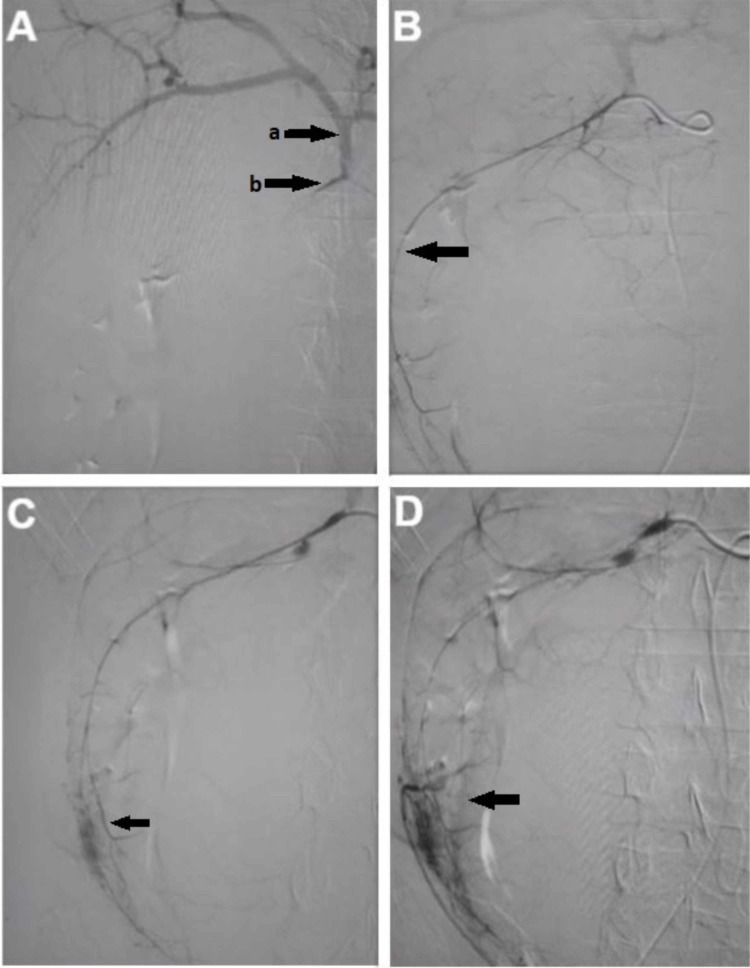
Serial images of attempted angioembolization of bleeding vessels of choledochal cyst during vascular intervention Figure 2A shows initial selective cannulation of gastroduodenal artery. a shows hepatic arterial system and b shows the origin of the gastroduodenal artery. Figure 2B shows the feeding vessel of the choledochal cyst arising from the gastroduodenal artery. Figures 2C, 2D show the blush representing bleeding into the choledochal cyst and confirming the diagnosis of arteriocholedochal fistula.

The patient was transferred to the vascular intervention suite after a brief period of resuscitation and hemodynamic stability as arteriocholedochal fistula was suspected. During angiography, selective cannulation of the gastroduodenal artery (GDA) showed bleeding into the cyst cavity (Figure 2). During the procedure, the patient again became hemodynamically unstable and also very restless. Due to the restlessness of the patient, cannulation of the GDA was lost with the artery going into spasm. Angioembolization was abandoned as multiple attempts to cannulate the GDA failed, and the patient’s condition was deteriorating. The patient was planned for emergency surgery and transferred to the operation theatre.

 

**Figure 3:**
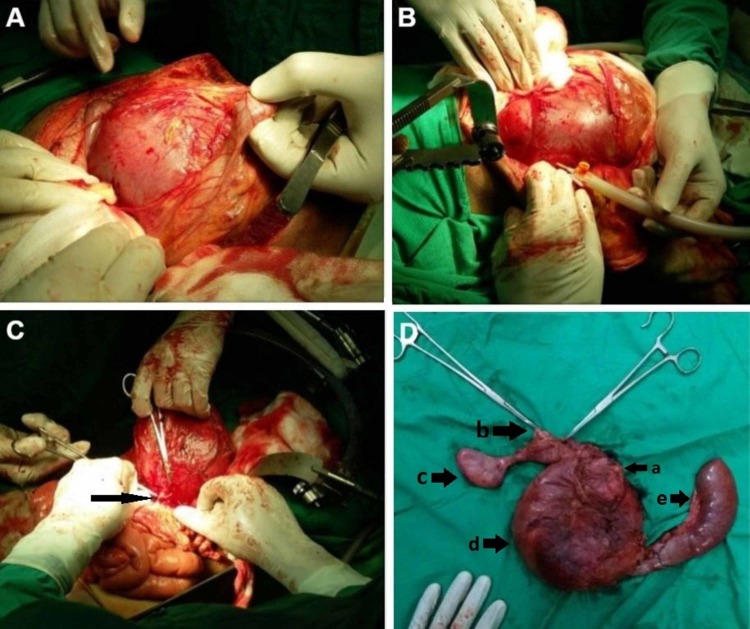
Peroperative pictures showing the giant choledochal cyst and final resected specimen Figure 3A shows the giant choledochal cyst upon opening the abdominal cavity. FIgure 3B shows aspiration of the giant choledochal cyst. Figure 3C shows the collapsed cyst with a feeding vessel being ligated that was arising from posterior to the pancreas, probably from a branch of the superior mesenteric artery. Figure 3D shows the final resected Whipple's pancreatoduodenectomy specimen. In Figure 3D, a shows the atrophied head of pancreas, b shows upper resection margin of the choledochal cyst, c shows the gallbladder and cystic duct, d shows collapsed choledochal cyst and e shows fourth part of the duodenum and jejunum

Exploratory laparotomy revealed a huge choledochal cyst extending into the right iliac fossa (Figure 3A). It was decided to undertake dissection at the hepatoduodenal ligament in order to identify the GDA and ligate it; however, as the large tense cyst was causing difficulty in exposure of this area, aspiration of the cyst was planned first to make the dissection less technically difficult. Approximately 2500 ml of blood mixed with bile was aspirated from the cyst through a small incision (Figure 3B). The cyst partially collapsed following aspiration of its contents. Following this, dissection was undertaken at the hepatoduodenal ligament. The GDA was identified at its origin from the common hepatic artery and was ligated. After ligation, a brief period of observation showed that the cyst was re-expanding, and the patient’s vital signs were not improving. Further blood was aspirated from the previously made small incision. It was inferred that there must be additional vessels bleeding into the cyst, possibly from the superior mesenteric artery. It was thus decided to perform Kocherization and identify these feeding vessels. Kocherization revealed that the large cyst densely adhered to the duodenum as if duodenum was part of the cyst wall. Feeding vessels were also found originating from posterior to the head of pancreas (Figure 3C). The head of the pancreas itself was severely atrophied. In view of the above findings, it was decided to proceed with a Whipple’s pancreatoduodenectomy in order to control bleeding and resect the cyst (Figure 3D). At the end of the resection, the patient’s vitals improved, and he remained stable during the anastomotic reconstruction. Anastomotic reconstruction was undertaken by using a Roux-en-Y limb (jejunum) with an end-to-end pancreatojejunostomy (in invaginated fashion), end-to-side hepaticojejunostomy and side-to-side gastrojejunostomy. A Braun jejunojejunostomy (an anastomosis between the afferent and efferent limb of the gastrojejunostomy) was also created as a measure to limit postoperative bilious vomiting, afferent limb syndrome and delayed gastric emptying. The patient remained stable postoperatively. After a three-day ICU stay, he was shifted to the ward.

The patient developed purulent discharge from his wound on the third postoperative day for which his wound was partially opened. He was discharged on the 10th postoperative day but re-admitted a day later due to upper gastrointestinal (GI) hemorrhage. This resolved spontaneously with conservative management. Endoscopy the next day revealed a non-bleeding ulcer with a clot at its base at the gastrojejunostomy site. The patient was discharged three weeks after the second admission in stable condition. There had been no further episodes of upper GI hemorrhage; however, he was re-admitted twice due to severe upper GI upset during the first few weeks following surgery. The histopathology report of the Whipple’s specimen revealed low-grade dysplasia in part of the cyst wall. However, no malignancy was identified.

## Discussion

Arteriocholedochal fistulas are unusual findings that have been commonly reported as an iatrogenic complication [4]. Giant choledochal cysts are also rare findings as choledochal cysts rarely exceed 9 cm in size [12]. However, an extensive literature search revealed that arteriocholedochal fistulas had not been reported to occur in giant choledochal cysts. Arteriocholedochal fistulas with life-threatening (major) hemobilia usually occur due to some pathology of vessels surrounding the biliary tract [1,5,6]. A pseudoaneurysm develops due to injury of vessels during an invasive procedure in this area or due to vasculitis [1]. With time an arteriocholedochal fistula is formed as the perivascular inflammatory process erodes and bursts into adjacent bile ducts, creating GI hemorrhage in the form of hemobilia [1,2]. Hemobilia has occurred after inflammation or infection of the biliary tract but represents cases of mild or minor hemobilia which are not life-threatening [1]. This case represents a unique situation in which pathology of the common bile duct led to a spontaneous arteriocholedochal fistula with life-threatening consequences. The arteriocholedochal fistula developed because the choledochal cyst got infected and inflamed. Choledochal cysts are known to develop complications such as spontaneous rupture, biliary peritonitis, and cholangitis, however, development of an arteriocholedochal fistula in its wall has not been reported [11,13,14]. It is proposed that inflammation of the cyst wall led to erosion of its feeding vessels by the following mechanism. Although the inflammatory process improved during the first week of admission with conservative management, the process probably amalgamated into severe transmural inflammation of part of the choledochal cyst wall that also led to inflammation and damage of the walls of its feeding vessels. The process of damage to the vessel wall continued over the first week until the whole vessel wall got eroded and bleeding started at the beginning of the second week of admission. Because the choledochal cyst in this patient was of a very large size, it had multiple feeding vessels from all surrounding major vessels, the duodenum, and pancreas. Thus, erosion of multiple vessels probably caused a significant amount of blood loss.

Hemobilia is recognized by the classical triad of right upper quadrant abdominal pain, jaundice, and GI bleeding [8]. However, it is not necessary that all three symptoms be present [8]. If the flow of blood into the duodenum through the biliary tract is blocked by blood clots, gastrointestinal bleeding may not occur, especially, if bleeding is not brisk [4,5,8]. Our patient also did not have GI bleeding as a clinical finding, possibly because of a blood clot at the outflow. Because of this, initially, blood could not escape in significant amount through the ampulla of the duodenum to become clinically evident as hematemesis or melena. As the cyst expanded suddenly to reach the larger size due to the accumulating blood, kinking of the biliary tract probably became a further hindrance to blood escaping into the duodenum even in the presence of brisk bleeding. The sudden increase in the size of the cyst that was appreciated clinically was the result of this series of events and is also a unique finding in this case. Although bleeding was contained within the expanding cyst to a certain extent, the loss of blood was enough to put the patient into class II hypovolemic shock, which combined with the possible ongoing element of sepsis, made his condition worse.

Management of patients with major hemobilia should involve emergent angiography and embolization of feeding vessels to stop bleeding [2,5,8]. Although this is the preferred treatment option, in case of failure, these patients are left with the option of surgical intervention [2]. Angioembolization was attempted in our patient; however, as the patient became very restless during the procedure, it could not be completed successfully. The restlessness of the patient did not allow him to lie down in one position, which led to the loss of cannulation of the GDA after it had been successfully negotiated, and the bleeding vessel was recognized. Sedation could not be given because of the chances of circulatory collapse, and for a similar reason, IV nitrates were not administered to overcome spasm of GDA that occurred after cannulation of the vessel was lost. It is also evident from this case that a giant choledochal cyst may have additional bleeding vessels that can be eroded because of the inflammatory process. Thus, even if angioembolization had been successful through the GDA, further angiogram of the superior mesenteric artery would have been required to identify and treat other possible eroded vessels. Extensive collaterals and technical difficulties are recognized causes of failure of vascular intervention [2].

Since choledochal cysts are pre-malignant, there is a universal agreement in favor of complete resection after they are diagnosed [11]. However, in cases where there has been repeated cholangitis and pericystic inflammation, dense adhesions can be expected with adjacent structures [12]. In such cases, partial endocystic resection of the cyst wall is recommended [12]. Unfortunately, our patient was planned for emergency surgery with the aim to stop bleeding and resection of the cyst as a secondary objective if conditions allowed. Per-operative findings revealed that there was continuous bleeding within the cyst from multiple vessels and that the cyst densely adhered to the duodenum and head of the pancreas. If it was decided that the cyst should not be removed and all possible feeding vessels be ligated from outside, this would have been a very tedious procedure and could also result in compromised blood supply of the duodenum and head of pancreas with postoperative delayed sequelae. Such a procedure could also lead to intraoperative damage to the duodenum and head of the pancreas, creating further problems during the surgery. While opening up the cyst to control bleeding from inside would have allowed endocystic resection of part of the cyst’s wall as well, it was not deemed possible because of chances of free bleeding into the peritoneal cavity with catastrophic consequences. Once we were inside the cyst, efforts aimed at stopping bleeding from these vessels would also be catastrophic as adjacent structures would be exposed to injury during rapid and aggressive attempts at securing hemostasis. Due to the above deliberation, it was decided to undertake WPD as this procedure would allow complete resection of the whole cyst and avoid unnecessary bleeding that was presently limited to within the cyst cavity because of tamponade effect of the cyst wall.

WPD is no doubt a major undertaking and requires a certain level of surgical expertise; this case represents a good example of why general surgeons should also train themselves in this procedure [15]. If expertise is not available, it is best practice that patients with giant choledochal cysts involving duodenum and head of pancreas be referred to subspecialty centers with adequate surgical expertise even if the patient is planned for elective resection. The reason is that per-operative findings may dictate that complete resection of the cyst is not possible due to densely adhered duodenum and pancreatic head as in this case. If the patient presents with cholangitis, an urgent referral is more warranted as bleeding into the inflamed cyst due to a spontaneous arteriocholedochal fistula may occur at any time.

## Conclusions

Arteriocholedochal fistula and major hemobilia can be a complication of a giant choledochal cyst if it becomes infected or inflamed. The first line of definitive treatment should be by vascular interventional radiology, where multiple bleeding vessels should be sought. WPD is a viable treatment option and should be decided upon early to avoid more deleterious per-operative complications of other surgical procedures; however, only when the required surgical expertise is available. In view of this, a high index of suspicion and contemplation of a possible series of events should warrant the involvement of surgeons trained in hepatopancreatobiliary surgery or urgent referral of such cases to hepatopancreatobiliary subspecialty centers.
